# Memory and Forgetting

**DOI:** 10.1007/s11920-018-0950-7

**Published:** 2018-08-28

**Authors:** Chris R. Brewin

**Affiliations:** Clinical Educational & Health Psychology, University College London, Gower Street, London, WC1E 6BT UK

**Keywords:** Trauma, PTSD, Reconsolidation, Retrieval, Imagery, Therapy

## Abstract

**Purpose of Review:**

I summarize recent developments in understanding the phenomenology of memory in PTSD, describe the most prominent theoretical models, and outline new forms of treatment aimed at modifying the traumatic memory.

**Recent Findings:**

Intrusive memories that have the quality of being relived in the present have been highlighted in ICD-11. Debate over whether trauma memories are disorganized has led to a distinction between global narratives that are usually well rehearsed and episodic memories of the most frightening moments when disruptions and fragmentation may occur. Attempts to prevent the initial consolidation of trauma memories have promise in prevention but face practical difficulties. Theoretical developments have led to a number of promising treatments for established PTSD including pre-retrieval propranolol and imagery rescripting.

**Summary:**

Research has suggested real possibilities to improve the prevention and treatment of PTSD by modifying trauma recall even though the theoretical basis for these interventions remains controversial.

## Introduction

The proposal that posttraumatic stress disorder (PTSD) is primarily a disorder of memory [1, 2] continues to gain traction. Reviews have confirmed that PTSD is associated with deficits in memory for emotionally neutral information that are stronger for verbal than visual materials [3]. These verbal memory deficits, as well as overgeneral autobiographical memory, avoidance or suppression of memories, and negative interpretation of memory symptoms, are likely to play a causal role in the development or maintenance of PTSD [4]. In addition, memories for the traumatic event itself are typically seen as altered in two distinct ways: There is impairment in the voluntary retrieval of these coupled with an increased incidence of a specific type of involuntary memory sometimes referred to as a “flashback” [4–6]. Contrary to widespread opinion, traumatic events are sometimes not well remembered and can be forgotten.

## Phenomenology

### Memory

Whereas it was originally believed that intrusive memories of unpleasant experiences were a unique symptom of PTSD, it is now known that this symptom is common in most psychiatric disorders [7]. What appears to distinguish the intrusive memories in PTSD is that they are experienced as though they were happening in the here and now [8–11]. This can be thought of as a dissociative alteration to the sense of time and is sometimes referred to as a “flashback.” DSM-5 [12] now clarifies that this symptom exists on a continuum from a brief sense of the event happening again in the present to a total absorption in the traumatic memory with loss of awareness of the current environment. The conceptualization of PTSD in ICD-11 [13, 14] has identified this specific form of re-experiencing (whether as part of intrusive memories, flashbacks, or nightmares) as required for the diagnosis.

There is general agreement amongst clinicians that traumatic memories are often disorganized and fragmented to some degree, consistent with the DSM-5 symptom involving inability to recall key features of the traumatic event [12]. Studies have consistently found that independent judges rate the narratives of PTSD patients as being more disorganized than both their own non-trauma narratives and the trauma narratives of individuals without PTSD [15]. Experimental evidence likewise confirms that negative affect has the effect of improving memory for central items while impairing memory for context [16•, 17] and makes representations stronger but less rich [18].

A number of studies have, however, failed to find such differences when comparing the narratives of individuals with and without PTSD [19]. These studies have generally used global ratings of the entire trauma narrative rather than the local focus on sections of text used by the clinical studies. This suggests that the discrepancies between the two sets of studies can be explained by methodological differences [20•]. Specifically, fragmentation and disorganization are likely to be associated with highly emotional moments during the traumatic event when cognitive processing is disrupted (e.g., by a dissociative response). In contrast, PTSD sufferers may be perfectly able to provide a general account to others of what happened to them that is rehearsed and coherent but that omits details of the worst moments of the trauma.

### Forgetting

Even though they may be highly distressing at the time, not all traumatic events become part of a person’s life story. Like other autobiographical memories, they are more likely to be remembered if they are part of a shared social experience or have personal consequences. Forgetting has been documented for individual traumatic experiences [21] and for repeated traumatic events such as childhood abuse [22]. In understanding how repeated events could be forgotten, there are various factors that need to be considered: How the events have been framed by the perpetrator or other family members, whether trauma has led to the development of a fragmented sense of self, and whether there have been deliberate attempts to forget the events [21]. There is abundant evidence that the deliberate attempts to forget mentioned by some abuse survivors are plausible in neurobiological terms. Suppressing the retrieval of an unwanted memory when a reminder to that memory appears is reliably associated with top-down modulation of hippocampal activity by the dorsolateral prefrontal cortex [23•].

When the trauma survivor is suffering from PTSD, however, attempts to forget are typically difficult or impossible. In the directed forgetting paradigm, participants are required to forget experimental items that have just been presented, and PTSD is associated with greater difficulty in doing this [24]. More recently, patients have been taught to associate aversive scenes with naturalistic reminders and then to practice voluntarily suppressing the scenes when cued with the reminders [25•]. This task assesses inhibitory control of memory retrieval, a skill extremely relevant to PTSD patients. The results indicated that retrieval suppression was compromised significantly in PTSD patients and that those with the largest deficits in suppression-induced forgetting were also those with the most severe symptoms. The authors suggested that the difficulties patients have in controlling their intrusive memories arise partly from deficits in engaging inhibitory control to suppress retrieval. This raises the possibility that therapeutic approaches which attempt to have patients confront, and then suppress, their traumatic memories might be a valuable adjunct to standard psychological treatment.

## Memory Models

### Fear Conditioning, Extinction, and Reconsolidation

The dominant memory model for neurobiological research has been that of fear conditioning, which lends itself to elegant animal experiments and neuroimaging studies. The model was originally designed to account for the adaptive acquisition and loss of associations between stimuli that predicted aversive or desired outcomes and various behavioral and physiological responses. A variety of more specific processes can be distinguished: associations formed to the aversive stimuli themselves, to their context, and to safety cues; the process by which cues related to the aversive stimuli come to evoke similar responses (generalization); the process by which subsequent non-reinforced presentations of the aversive stimuli lead to the weakening of the acquired association (extinction); and the process by which after extinction the associations may come to be expressed once again either spontaneously or through exposure to appropriate reminders (renewal and reinstatement).

Psychotherapy for PTSD is essentially a process of altering the nature or expression of what has been learned during the traumatic event. There has been a long-standing debate in several areas of psychology between proponents of the idea that new learning overwrites the original memory and changes it permanently, and those who consider that new learning cannot overwrite the past but instead creates an alternative memory that then competes with the original. It is thought that extinction learning leads to the formation of new, potentially inhibitory, memories that pair the aversive stimuli with safety or with an absence of punishment, and that in the presence of reminders the original and new sets of memories compete for retrieval.

In addition to the extinction mechanism, it was initially proposed that each time memories are brought to mind, they need to be reconsolidated, and that this provides an opportunity for the memory to be permanently altered by the incorporation of new information or even erased [26]. Animal experiments sought to first teach an association and then demonstrate that following a reminder of the prior learning administration of a protein synthesis blocker led to the memory no longer being expressed. As such, drugs are restricted to animal use, human experiments typically paired an aversive event such as shock with a neutral stimulus to create a conditioned emotional response, and then manipulated whether or not the participant received a reminder of the stimulus prior to an intervention such as an anxiolytic drug or an extinction procedure. Demonstrating that the presence of the reminder significantly moderated the effectiveness of the intervention was taken as support for the reconsolidation hypothesis. Such procedures have been used to decrease fear expression, as indexed by a startle response, while leaving the declarative memory of the learning event intact [27, 28].

Subsequent research has not always been able to replicate these findings [29, 30]. When they are effective, such procedures appear to affect conditioned emotional reactions but not conscious appraisals. There are indications that reconsolidation is harder to obtain with stronger and more remote memories, or with more anxious participants, and that reminders do not invariably trigger the reconsolidation process. Rather, to be effective, reminders need to signal a surprising or unpredicted event [31••]. In addition to these limitations, there has been debate over whether the procedures that stand in for protein synthesis blockers have been able to conclusively demonstrate reconsolidation in humans [32, 33].

Underlying these mechanisms are a number of neural circuits. Threat detection involves the amygdala as a key structure that receives input signaling threat and orchestrates a number of pre-programmed responses. The amygdala is regulated by top-down control from the medial prefrontal cortex, with the dorsal anterior cingulate cortex being involved in the expression of fear and the ventromedial region of the prefrontal cortex being involved in the recall of fear extinction. Another circuit, involving the amygdala, medial prefrontal cortex, and hippocampus, is concerned with the processing of context, enabling what has been learned about threat to be expressed or inhibited depending on the prevailing conditions [34].

Although currently symptomatic individuals do often respond differently to controls in tests of fear conditioning, this may be a marker of existing disorder rather than a causal element. The fact that only a minority of individuals develop pathological responses after exposure to traumatic events has led to proposals that this minority may be characterized by deficits in some of the specific processes underlying fear learning, such as contextual conditioning or extinction learning. Studies showing disturbances in aversive learning prior to the development of clinical disorders are rare. There is also relatively little evidence that known vulnerability factors for PTSD are related to dysfunctional fear-learning patterns in non-clinical populations [35]. Another limitation of the fear conditioning model is that it does not address the intrusive autobiographical recall of the traumatic event and the negative appraisals that are such a prominent part of the experience of PTSD.

### Dual Representation Theory

Other models are situated more directly within research on autobiographical memory and address the predominantly visual nature of re-experiencing. According to the revised dual representation theory of PTSD [7, 15], flashbacks depend on a stress-related excess of activity in the dorsal visual stream, which is specialized for creating images of the environment from a first-person (egocentric) perspective that can be used to direct immediate motor responses to threat. Very high levels of stress also lead to a corresponding reduction of activity in the ventral visual stream and medial temporal lobe, where the elements of objects and scenes are normally bound together and encoded in an abstract form. Under normal conditions, this abstract coding enables objects and scenes to be identified, manipulated, imagined from alternative (allocentric) perspectives, and related to past experience, forming the basis for higher-order cognitive appraisal. The result of extreme stress is poorly contextualized, fragmented images and scenes that when triggered by trauma reminders are experienced as flashbacks. Consistent with this model, PTSD patients appear to have a selective deficit in allocentric spatial memory, implicating weaker hippocampal functioning [36]. In this study and in a separate study of navigation [37], exposure to previous trauma was associated with a greater impairment in specific aspects of spatial processing.

Despite their importance, the differences between flashbacks and ordinary episodic memories of the same traumatic event have been relatively little studied. As previously summarized [38], neuroimaging studies have found flashbacks to be associated with increased activation in sensory and motor areas including the insula, precentral gyrus, supplementary motor area, and mid-occipital cortex, but with decreased activation in a medial temporal area, the parahippocampal gyrus. Patients reporting more flashbacks also appear to have reduced brain volume in areas of the visual ventral stream. Like the real-life situations PTSD patients encounter, the same words and phrases tend to elicit flashbacks repeatedly, but not invariably—flashback elicitation is a probabilistic rather than a wholly predictable process. A recent study of the voluntary recall of trauma memories by a PTSD sample, mimicking what happens in exposure therapy, found that whereas heart rate gradually reduced over time, flashbacks were accompanied by momentary increases in heart rate [39].

## Preventive and Treatment Interventions Aimed at Traumatic Memories

### Prevention

Based on the idea that overconsolidation of the traumatic memory is causally involved in PTSD, several studies have attempted to block consolidation by giving the beta-adrenergic receptor antagonist propranolol to patients admitted to the emergency room in the hours immediately following a traumatic event. A meta-analytic review of five studies failed to demonstrate an overall effect on reducing the severity of PTSD symptoms [40]. Despite these negative results, it is important to note propranolol is not fully absorbed for 60–90 minutes and that most studies did not succeed in achieving adequate propranolol concentrations within the 6-hour window within which most consolidation is thought to occur. The studies also faced considerable difficulties in recruitment. Thus, although the proposed mechanism has not yet been fully tested, practical considerations mean that this intervention is unlikely to make a substantial impact in practice.

An alternative to pharmacological intervention is the use of competing tasks that are hypothesized to interfere with consolidation of the sensory aspects of the trauma memories. A recent study [41•] compared a trauma reminder followed by 20 minutes playing a computer game with high visuospatial demands (Tetris) with an attention-placebo control in patients admitted to an emergency department within six hours of a motor vehicle accident. Patients who played Tetris reported finding this easy and helpful. They experienced fewer intrusive trauma memories in the subsequent week but did not differ on other symptoms of PTSD or on any symptom at one month. The absence of significant differences at one month reflected improvement in the control group rather than any return of trauma memories in the Tetris group. The study was limited by the low overall rate of patients developing PTSD post-accident and was unable to determine whether the initial trauma reminder was an essential element of the intervention, but suggested that further trials with larger and more severely affected samples are warranted.

### Treatment

Most recognized forms of psychological treatment for PTSD, such as trauma-focused cognitive-behavior therapy, eye movement desensitization and reprocessing (EMDR), and narrative exposure therapy, involve overcoming avoidance and bringing about a degree of exposure to the traumatic memory, with a focus on addressing the most intense or painful moments likely to be particularly helpful [42]. This can often be achieved in as little as five sessions lasting in total less than four hours [43••]. Although earlier methods often used quite prolonged exposure to the traumatic memory, more recent approaches, including EMDR and imagery rescripting [44, 45•], involve relatively brief exposure with an emphasis on incorporating new elements into the traumatic image or imagining the scene from a different perspective. These interventions can lead to a rapid reduction of fear and other negative emotions.

A number of theoretical mechanisms have been implicated in exposure therapy and other psychological therapies for PTSD. One prominent rationale for prolonged exposure suggested the mechanism was initial fear activation followed by habituation of fear within and between treatment sessions [46], but the evidence for this is weak [47, 48]. Consistent with the inhibitory view of extinction learning and the dual representation theory of PTSD, the success of the newer therapies such as EMDR or imagery rescripting suggests that a critical mechanism in PTSD therapies is the contextualization of the traumatic memory by having the person deliberately focus attention upon it in a safe environment or introduce safe elements into the image. This may include methods of contextualization that are known to increase hippocampal activity such as imagining the scene from an alternative perspective [49]. The recontextualized memory, in which the trauma is now located in a safer temporal and spatial context, may act as an inhibitory version of the original traumatic memory [50], which is now available to compete with the original traumatic memory. Retrieval competition and mutual inhibition between relatively aversive and benign versions of key life events are thought to be general mechanisms underlying the success of cognitive-behavior therapy [51].

An alternative to these mechanisms is provided by the reconsolidation hypothesis, which has led to interventions for several different clinical populations, with moderate levels of success [52]. One of the most important considerations is that the procedures must not only lead to memory retrieval but must successfully destabilize it and then update it with incompatible information [53]. One intervention that has met with some success is to administer propranolol to PTSD patients before they recall their trauma. This allows them to experience the memory in a new way, with much reduced physiological and emotional arousal. In a recent clinical trial [54••], patients wrote a one-page trauma narrative focusing on the event’s most disturbing moments 60–90 minutes post-propranolol or placebo. This narrative task took up to 30 minutes, after which the patient read the narrative aloud once to the therapist “as if they were back in the event.” Over the course of six weekly sessions, the propranolol group showed a substantial improvement in symptoms relative to the placebo group.

Another open-label single-case series has trialed a novel intervention for complex PTSD based on the competing task rationale described above [55•]. Twenty patients in inpatient treatment monitored the occurrence of intrusive trauma memories over the course of their admission. Weekly interventions involved targeting a selected memory (typically one that had been particularly distressing), writing a brief narrative about it, and then immediately spending 25 minutes playing Tetris. The frequency of targeted intrusive memories reduced by on average 64% from baseline to the post-intervention phase, whereas never-targeted intrusions reduced in frequency by on average 11% over a comparable time period. For targeted intrusions, reduction in intrusion frequency was significantly positively correlated with reduction in measures of depression and anxiety.

There are, however, numerous difficulties in applying the reconsolidation hypothesis to clinical practice. Confronting traumatic memories in psychotherapy is not like laboratory extinction methods in which a single non-reinforced CS+ is repeatedly presented. The traumatic memory is a complex experience consisting of multiple stimuli and responses, linked to meanings derived from the event as well as associated past experiences. It is difficult if not impossible to recreate the circumstances of the original event and simply change the CS-UCS contingencies. For these reasons, exposure therapy is often thought to rely on other processes such as habituation [46], UCS revaluation [56], or contextualization [7].

Standard exposure therapy involves repeated generation of prediction errors as the patient is encouraged to identify and hold in mind aspects of the traumatic memory that have previously appeared too aversive to tolerate. The focus on deliberate, detailed recall, particularly of the most frightening moments, tends to generate spontaneous flashbacks that contain sensory images encoded during the traumatic event. These procedures are thought to lead to the formation of new contextualized memories containing the information that the negative affect associated with trauma reminders is more short-lived or less intense than expected. As noted by Kindt (2018), if the aim of exposure is instead to produce memory updating and reconsolidation, it should be much shorter and occur only once.

In Brunet et al.’s (2018) study, there was extended exposure to the traumatic memories on multiple occasions, raising the possibility that the procedures were effective because they led to enhanced extinction rather than to memory reconsolidation [57]. Kessler et al.’s (in press) procedures did use briefer exposures that targeted specific episodes within the overall traumatic memory, but it is unclear how these exposures would have generated prediction errors. Demonstrating that it is specifically reconsolidation that is being affected is likely to be an issue with most clinical studies, because patients will arrive with many of the details of their troubling memories already on their mind, if not actively intruding prior to or during the therapy session. In other words, their existing mental state will make it very difficult to manipulate the “reactivation” of the trauma memory that is necessary to support the reconsolidation hypothesis. Moreover, “reactivation” is likely to be associated with involuntary intrusion of the memories, and it may be this additional element that, in combination with interventions such as propranolol or Tetris, is important for therapeutic success.

## Conclusions

PTSD is a complex disorder that may involve profound changes to the sense of self and to appraisal of others, the world, and the future. It is therefore surprising that interventions attempting primarily to reduce the intensity or frequency of intrusive trauma memories, such as exposure therapy, imagery rescripting, or memory recall under the influence of propranolol, are often effective. The implication is that the intrusive memories, along with the associated avoidance and hyperarousal, maintain negative appraisals and that, for some patients at least, blocking them or reducing their impact can restore pre-existing appraisals that were more positive.

In the search for new ways to block trauma memories, biological studies on memory consolidation and reconsolidation have generated a great deal of excitement. There have been several attempts to interfere with the initial consolidation of the traumatic memory, and thereby prevent the development of PTSD, but the practicality and effectiveness of these remains inconclusive. In contrast, the reconsolidation hypothesis has led to the use of propranolol combined with retrieval of the traumatic memory in patients with established PTSD, producing very promising outcome data, as well as to more preliminary work using competing tasks such as Tetris. Although early talk of “erasing” trauma memories now seems misleading, the opportunity to reduce physiological response to trauma reminders and to reduce the frequency and intensity of intrusive trauma memories is of considerable value. At present, however, despite a large number of findings consistent with the reconsolidation hypothesis, it is not possible to attribute therapeutic gains to this mechanism or even to conclusively infer that reconsolidation has been demonstrated in humans [51, 58]. Propranolol-induced reduction of arousal, for example, may be important for other reasons, such as enhancing extinction.

Research on the phenomenology of traumatic memories has reiterated the important distinction between disturbances in more global and rehearsed memory narratives and in the episodic account of the most frightening moments. At the more global level, disturbances have to do with difficulty in reconciling the facts of the traumatic event with individuals’ expectations of themselves and, for military veterans in particular, their expectations of others [59]. Traumatic events do not invariably involve fear but may involve loss or “moral injury,” the result of violation of accepted standards of behavior by oneself or others [60]. These different kinds of event are associated with different patterns of symptoms that may respond to standard exposure therapy but may additionally require more cognitive interventions.

At the more local level, disturbances in episodic memory associated with the most frightening moments of the trauma and with reactions such as dissociation are increasingly targeted within different types of exposure therapy. There are several innovative interventions, such as imagery rescripting and viewing the scene from other perspectives, that may generate alternative representations that are particularly effective at competing with and inhibiting the initial associations. This may be because in addition to reducing arousal, they are able to address critical aspects of cognitive appraisal such as powerlessness, loss of a sense of trust and safety, or abandonment by others. Such interventions are promising but as yet research is at a very early stage.

Theoretical models of memory and forgetting in PTSD need to consider lower-level associative learning, higher-order cognitive appraisals, and systems of meaning that can incorporate a social dimension, how multiple types of representation interact to influence responding, and how problematic representations can be made less accessible by processes such as UCS revaluation, interference, or retrieval suppression. Models should aspire to address the most prominent symptoms of PTSD, including how the traumatic scene is experienced visually. In translating these models into interventions, there is much to learn about the relative value of interfering with consolidation and reconsolidation as opposed to teaching retrieval suppression or constructing alternative inhibitory memories. Better understanding of the effects of trauma on memory will nevertheless likely be the key to effectively treating PTSD.
